# Supplementary figures and images for: Just Noticeable Difference Model for Images with Color Sensitivity
Source: Sensors (Basel). 2023 Feb 27;23(5):2634. doi: 10.3390/s23052634 (PMC10007073; doi:10.3390/s23052634)

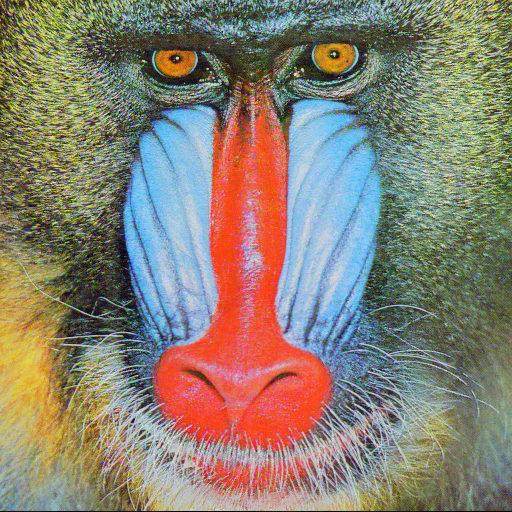

Supplement: Supplementary file 1 [file sensors-23-02634-s001.zip › supplementary file/4.2.03-Chen2019-1.27-20.571.png]

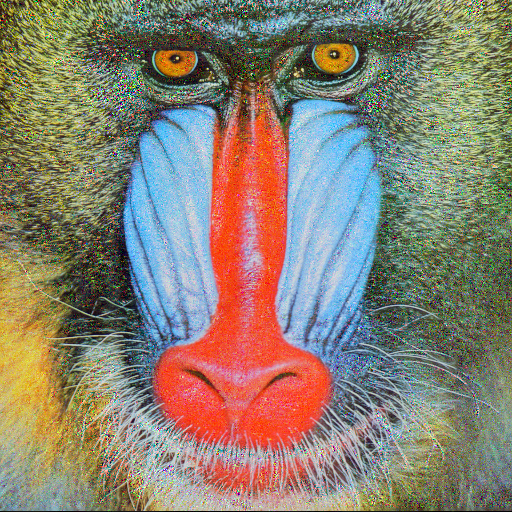

Supplement: Supplementary file 1 [file sensors-23-02634-s001.zip › supplementary file/4.2.03-Jiang2022-20.508.png]

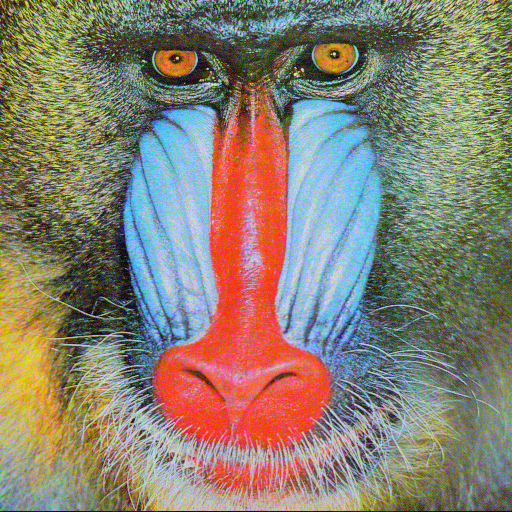

Supplement: Supplementary file 1 [file sensors-23-02634-s001.zip › supplementary file/4.2.03-propose-3.954-20.524.png]

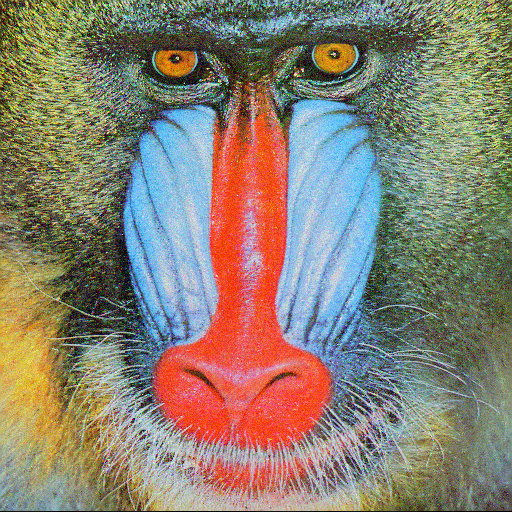

Supplement: Supplementary file 1 [file sensors-23-02634-s001.zip › supplementary file/4.2.03-Wu2013-1.803-20.527.png]

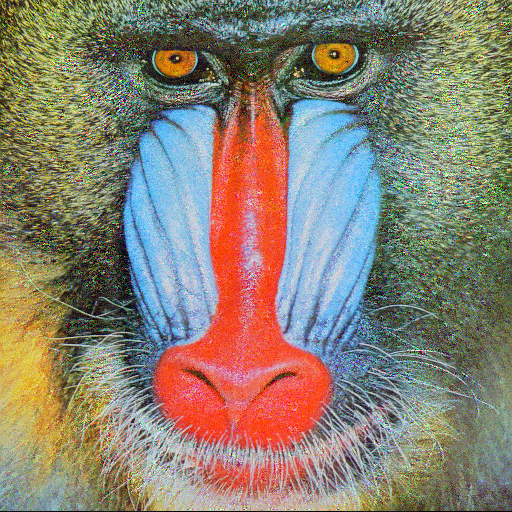

Supplement: Supplementary file 1 [file sensors-23-02634-s001.zip › supplementary file/4.2.03-Wu2017-1.5-20.526.png]

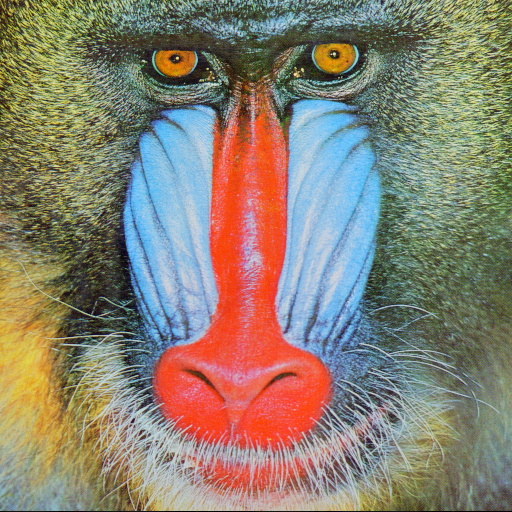

Supplement: Supplementary file 1 [file sensors-23-02634-s001.zip › supplementary file/4.2.03.tiff]

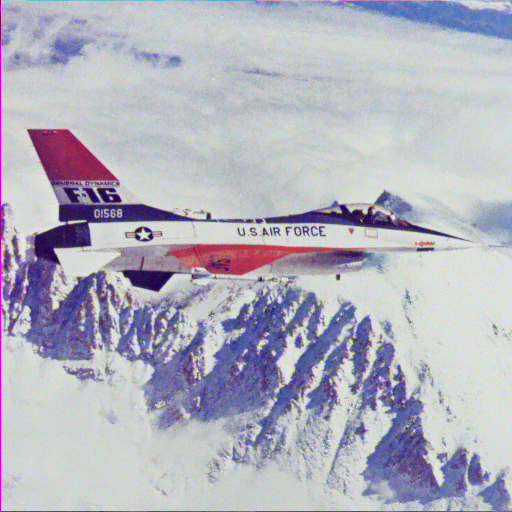

Supplement: Supplementary file 1 [file sensors-23-02634-s001.zip › supplementary file/4.2.05-Chen2019-1.5-26.550.png]

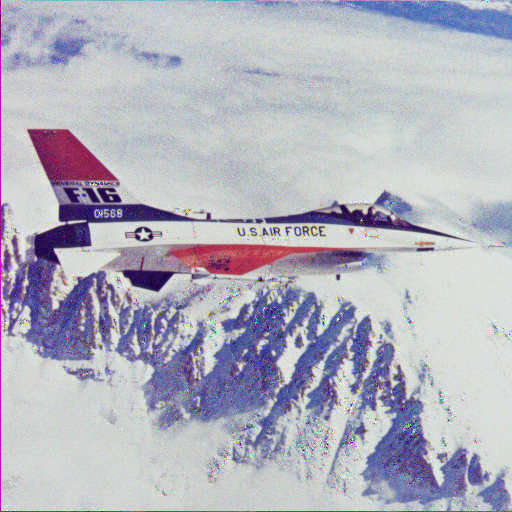

Supplement: Supplementary file 1 [file sensors-23-02634-s001.zip › supplementary file/4.2.05-Jiang2022-26.576.png]

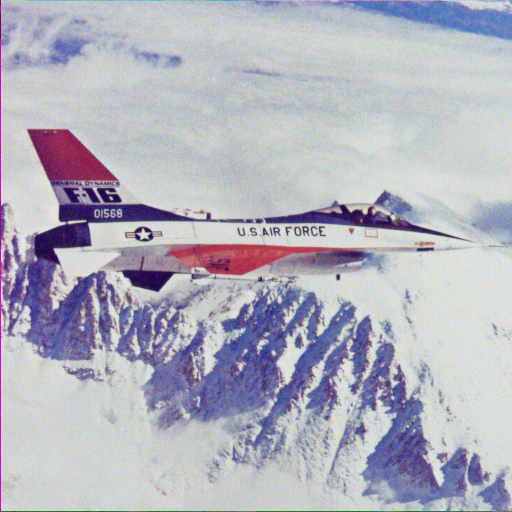

Supplement: Supplementary file 1 [file sensors-23-02634-s001.zip › supplementary file/4.2.05-propose-2.38-26.476.png]

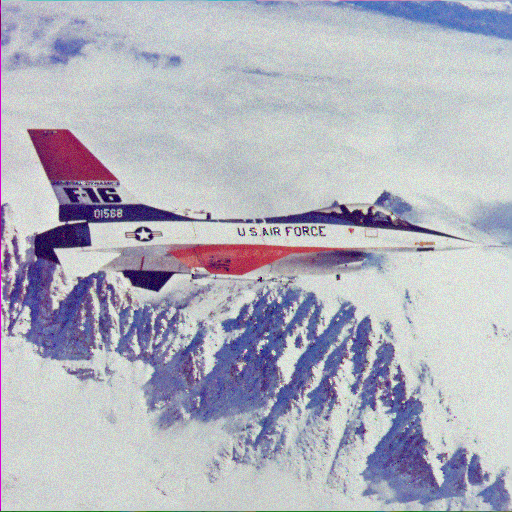

Supplement: Supplementary file 1 [file sensors-23-02634-s001.zip › supplementary file/4.2.05-Wu2013-1.573-26.474.png]

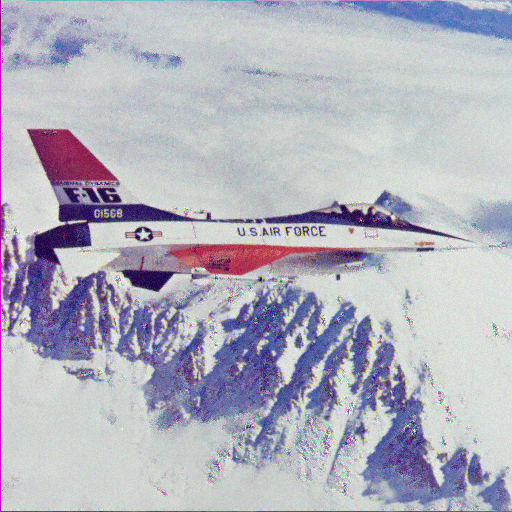

Supplement: Supplementary file 1 [file sensors-23-02634-s001.zip › supplementary file/4.2.05-Wu2017-1.5-26.481.png]

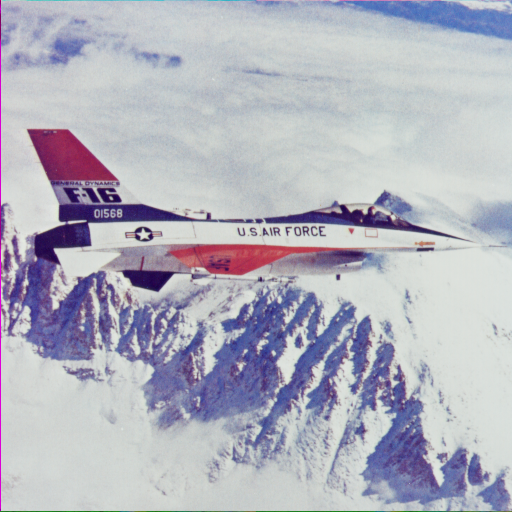

Supplement: Supplementary file 1 [file sensors-23-02634-s001.zip › supplementary file/4.2.05.tiff]

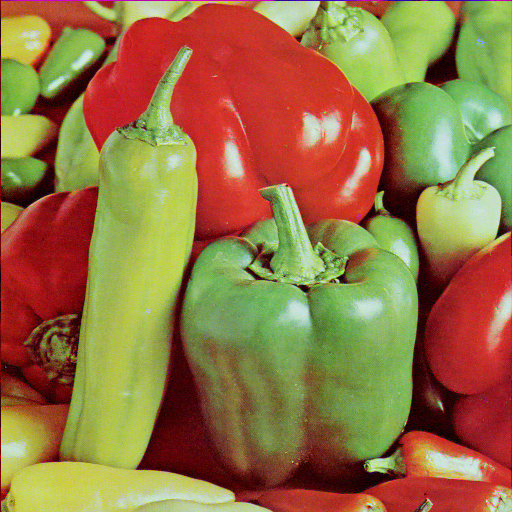

Supplement: Supplementary file 1 [file sensors-23-02634-s001.zip › supplementary file/4.2.07-Chen2019-1.19-26.103.png]

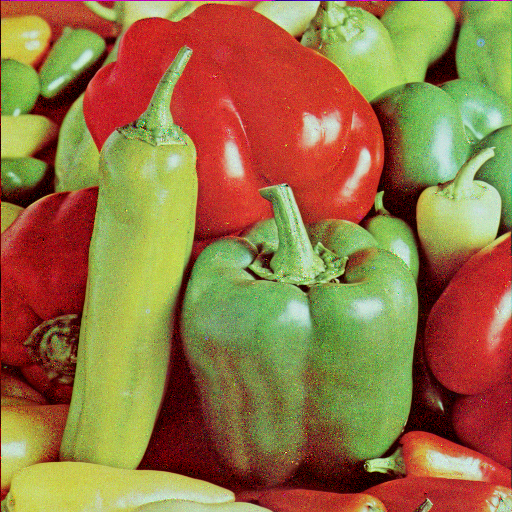

Supplement: Supplementary file 1 [file sensors-23-02634-s001.zip › supplementary file/4.2.07-Jiang2022-26.207.png]

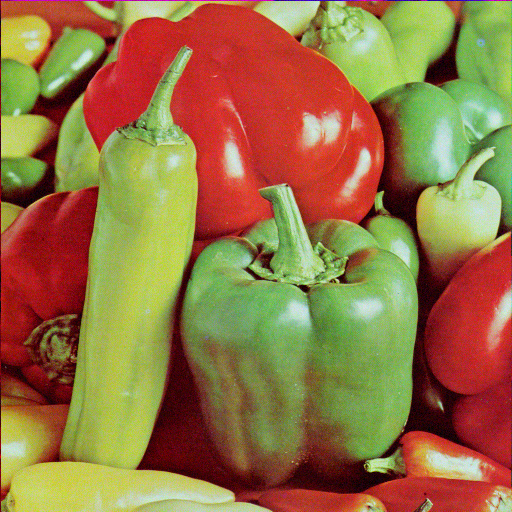

Supplement: Supplementary file 1 [file sensors-23-02634-s001.zip › supplementary file/4.2.07-propose-1.88-26.173.png]

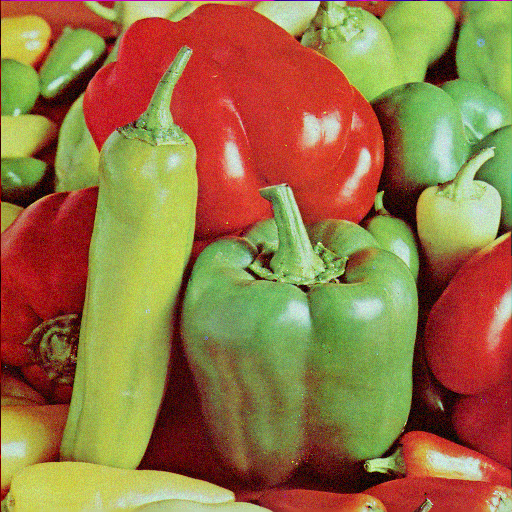

Supplement: Supplementary file 1 [file sensors-23-02634-s001.zip › supplementary file/4.2.07-Wu2013-1.421-26.178.png]

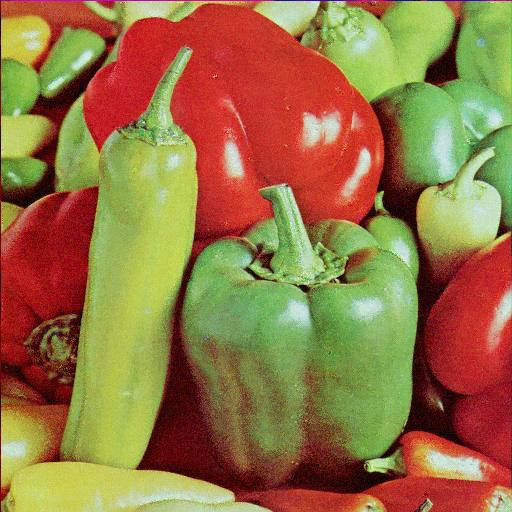

Supplement: Supplementary file 1 [file sensors-23-02634-s001.zip › supplementary file/4.2.07-Wu2017-1.5-26.174.png]

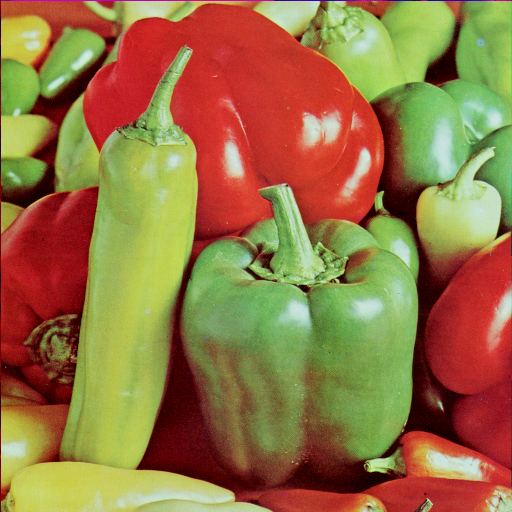

Supplement: Supplementary file 1 [file sensors-23-02634-s001.zip › supplementary file/4.2.07.tiff]
